# Mepolizumab has clinical benefits including oral corticosteroid sparing irrespective of baseline EGPA characteristics

**DOI:** 10.1183/23120541.00509-2023

**Published:** 2024-01-08

**Authors:** David R.W. Jayne, Benjamin Terrier, Bernhard Hellmich, Paneez Khoury, Lee Baylis, Jane H. Bentley, Jonathan Steinfeld, Steven W. Yancey, Namhee Kwon, Michael E. Wechsler, Praveen Akuthota

**Affiliations:** 1 Department of Medicine, University of Cambridge, Cambridge, UK; 2 Service de Médecine Interne, Hôpital Cochin, Paris, France; 3 Klinik für Innere Medizin, Rheumatologie und Immunologie, Medius Kliniken, Universität Tübingen, Kirchheim-Teck, Germany; 4 Human Eosinophil Section, Laboratory of Parasitic Diseases, National Institute of Allergy and Infectious Diseases, National Institutes of Health, Bethesda, MD, USA; 5 Global Medical Affairs, GSK, Durham, NC, USA; 6 Clinical Statistics, GSK, Brentford, UK; 7 Respiratory Therapy Area Unit and Flexible Discovery Unit, GSK, Philadelphia, PA, USA; 8 Respiratory Therapeutic Area, GSK, Research Triangle Park, NC, USA; 9 Clinical Sciences, Respiratory, GSK, Brentford, Middlesex, UK; 10 Department of Medicine, National Jewish Health, Denver, CO, USA; 11 Division of Pulmonary, Critical Care, and Sleep Medicine, University of California San Diego, La Jolla, CA, USA; 12 For a list of the members of the EGPA mepolizumab study team see the Acknowledgements

## Abstract

**Background:**

The Mepolizumab in Relapsing or Refractory EGPA (MIRRA) trial (GSK ID: 115921/NCT02020889) demonstrated that mepolizumab increased remission time and reduced oral corticosteroid (OCS) use compared with placebo in patients with relapsing or refractory eosinophilic granulomatosis with polyangiitis (EGPA). The present analysis investigated the impact of baseline characteristics on clinical outcomes and characterised the OCS-sparing effect of mepolizumab.

**Methods:**

In a phase 3, randomised controlled trial for patients with EGPA (MIRRA), patients received standard of care plus mepolizumab 300 mg or placebo every 4 weeks for 52 weeks. The accrued duration of remission, the proportion of patients in remission at weeks 36 and 48, and the proportion of patients with clinical benefit (remission, OCS or relapse-related) were assessed according to baseline EGPA characteristic subgroups (*post hoc*). Mepolizumab-related OCS-sparing benefits were also quantified.

**Results:**

Accrued duration of remission and the proportion of patients in remission at weeks 36 and 48 were greater with mepolizumab than placebo across the baseline subgroups of refractory disease, immunosuppressant use, EGPA duration, relapse number and OCS use ≤20 mg·day^−1^. The proportion of patients with clinical benefit was greater with mepolizumab *versus* placebo (range 76–81% *versus* 25–39%), irrespective of immunosuppressant use or EGPA duration. Patients treated with mepolizumab *versus* placebo accrued significantly more weeks on OCS ≤4 mg·day^−1^ (OR 5.06, 95% CI 2.47–10.38) and had a mean of 1423.1 mg less per-patient OCS exposure over 52 weeks.

**Conclusions:**

Mepolizumab treatment provided benefits to patients with EGPA across varying baseline clinical characteristics and can be considered an OCS-sparing treatment in EGPA.

## Tweetable abstract

**In *post hoc* analyses of a phase 3 trial, more patients with EGPA receiving mepolizumab *versus* placebo had clinical benefits, regardless of disease and treatment history. Mepolizumab can be considered an OCS-sparing treatment for patients with EGPA.** https://bit.ly/3QhBFpC

## Introduction

Eosinophilic granulomatosis with polyangiitis (EGPA) is a rare, progressive, systemic inflammatory disorder characterised by asthma, blood and tissue eosinophilia and vasculitis of small and medium vessels [1, 2]. Manifestations of EGPA are heterogenous, and can include weight loss, fever, myalgia, neuropathy, pulmonary infiltrates and/or sino-nasal abnormalities [3]. EGPA can be classified as a form of antineutrophil cytoplasmic antibody (ANCA)-associated vasculitis [1, 2], although only approximately 30–40% of patients with EGPA are ANCA-positive [4, 5]. The pathogenesis of EGPA is not fully elucidated; however, the prominent role of interleukin (IL)-5 in driving type 2 inflammation has been clearly demonstrated [6, 7].

Current management of EGPA focuses on induction and maintenance of remission [8, 9]. Oral corticosteroids (OCSs) may be used to induce remission [8, 9]; however, relapses are common and many patients require chronic OCS therapy [10, 11]. The risks of corticosteroid-associated adverse events increase with higher doses, and may contribute to patient morbidity, disease burden and reduced quality of life [12–16]. For patients with relapsing or refractory disease, non-corticosteroid-based immunosuppressants can be used for the induction of remission, for relapse prevention and to reduce corticosteroid use [9], although evidence for their efficacy in these respects is limited.

Mepolizumab is a humanised monoclonal antibody with proven treatment benefits across a range of eosinophil-driven diseases [17]. By targeting IL-5, the primary cytokine responsible for promoting eosinophil differentiation, activation and survival, mepolizumab reduces eosinophil counts, usually to within the physiological range [18–20]. The phase 3 Mepolizumab in Relapsing or Refractory EGPA (MIRRA) study demonstrated the safety and efficacy of mepolizumab in patients with EGPA. Patients receiving mepolizumab had an increased duration of remission and more patients achieved remission at weeks 36 and 48, compared with placebo [21]. Mepolizumab treatment resulted in a longer time to first relapse and reduced daily OCS dosage compared with placebo [21]. In addition, a *post hoc* analysis of the MIRRA study demonstrated further clinical benefits of mepolizumab, including more patients achieving ≥50% reduction in OCS dose over weeks 48 to 52 of the study period compared with placebo [22]. On the basis of the MIRRA study results, mepolizumab was approved for the treatment of EGPA in multiple countries [23–25].

Mepolizumab has proved to be an important treatment option for patients with EGPA in real-world clinical practice [26–28]. However, predictors of response to mepolizumab in patients with EGPA are not well established and although an OCS-sparing effect was demonstrated in MIRRA, this important treatment benefit was not described in detail. The aims of the current *post hoc* analyses of MIRRA data were to assess the impact of baseline clinical characteristics on the response to mepolizumab and to quantify and characterise the pattern of OCS-sparing effects of mepolizumab in patients with EGPA.

## Materials and methods

### Patients

MIRRA (GSK ID: 115921/NCT02020889) enrolled patients ≥18 years of age with a diagnosis of relapsing or refractory EGPA ≥6 months prior to the study. EGPA was defined by a history or presence of asthma, blood eosinophil count >1000 cells·μL^−1^ or >10% leukocytes, and two further features of EGPA (histopathological evidence of eosinophilic vasculitis, neuropathy, pulmonary infiltrates, sino-nasal abnormality, cardiomyopathy, glomerulonephritis, alveolar haemorrhage, palpable purpura or ANCA positivity). Patients with granulomatosis with polyangiitis or microscopic polyangiitis at screening were excluded, as were those who had organ-threatening or life-threatening EGPA ≤3 months before screening. Eligible patients were receiving a stable dose of prednisolone (≥7.5 to ≤50 mg·day^−1^, with or without stable immunosuppressants) for ≥4 weeks before baseline. Further details of patient eligibility criteria have been previously described [21].

### Study design

The design of MIRRA has been described in detail previously [21]. Briefly, MIRRA was a phase 3, randomised, double-blind, placebo-controlled, parallel-group, multicentre study of mepolizumab in patients with relapsing or refractory EGPA. Following a 1–4-week run-in period, patients were randomised (1:1) to receive mepolizumab 300 mg subcutaneously or placebo, plus standard of care (OCS, with or without immunosuppressants), every 4 weeks for 52 weeks. OCS dose remained stable between baseline (randomisation) and week 4, following which it could be tapered according to a standardised schedule. Immunosuppressant use remained stable throughout the study (if applicable).

### *Post hoc* end-points and assessments

These *post hoc* analyses of the MIRRA study assessed the co-primary end-points in MIRRA stratified by baseline clinical characteristics. The total accrued duration of remission (categorised as 0 weeks, 1–11 weeks, 12–23 weeks, 24–35 weeks, ≥36 weeks) and proportion of patients in remission at weeks 36 and 48 were stratified by baseline refractory disease status (with/without), baseline immunosuppressant use (with/without), EGPA duration (≤4/>4 years, defined based on the median disease duration), number of relapses in the previous 2 years (0, 1–2, ≥3) and baseline OCS dose (≤7.5 mg·day^−1^, >7.5–≤12 mg·day^−1^, >12–≤20 mg·day^−1^, >20 mg·day^−1^). Additionally, patients who achieved clinical benefit, defined as 1) remission at any point during the 52-week treatment period and/or 2) ≥50% reduction from baseline in OCS dose during weeks 48–52 and/or 3) no relapses during the treatment period, were stratified by baseline immunosuppressant use and EGPA duration.

For the analyses described above, remission was defined as Birmingham Vasculitis Activity Score (BVAS)=0 and prednisolone/prednisone dose ≤4 mg·day^−1^. Relapse was defined as 1) BVAS>0 (active vasculitis), 2) worsening Asthma Control Questionnaire (ACQ)-6 score from most recent previous assessment or 3) worsening sino-nasal symptoms from most recent previous assessment (symptoms of runny nose, post-nasal discharge, facial pain/pressure, loss or reduction in sense of taste/smell and blockage/congestion of nose were rated weekly by patients using an eDiary) leading to 1) an increase in prednisolone/prednisone dose to >4 mg·day^−1^, 2) initiation of/increase from baseline in immunosuppressant therapy dose or 3) hospitalisation. Baseline refractory disease within 6 months prior to screening was defined as either 1) failure to attain BVAS=0 and OCS ≤7.5 mg·day^−1^ following ≥3 months of remission induction treatment with a standard regimen or 2) recurrence of EGPA symptoms while tapering OCS occurring at an OCS dose ≥7.5 mg·day^−1^.

In addition, the following OCS (prednisolone/prednisone)-sparing end-points were assessed *post hoc*: the proportion of patients with >50% reduction from baseline in OCS during weeks 48–52 stratified by baseline immunosuppressant use and EGPA duration; the proportion of patients receiving OCS ≤4 mg·day^−1^ and ≤7.5 mg·day^−1^ at weeks 36 and 48; accrued weeks of OCS ≤4 mg·day^−1^; accrued OCS-free weeks (OCS=0 mg·day^−1^); mean OCS daily dose during weeks 48–52; and cumulative OCS exposure over the treatment period.

### Sample size and statistical analysis

Sample size calculations for the primary and secondary outcomes of the MIRRA study have been described previously [21]. In the current analysis, ordered categorical data were analysed using proportional-odds regression. Binary outcomes were analysed using logistic regression. Patients who discontinued mepolizumab or placebo were followed until the end of the study, when possible, and all efficacy data were included in the analysis. All data were analysed using SAS version 9 (SAS Institute, Cary, NC, USA).

## Results

### Patient population

The MIRRA intent-to-treat population included 136 patients; 68 received mepolizumab and 68 received placebo, in addition to standard of care. Demographics and baseline characteristics for both mepolizumab and placebo groups are shown in table 1 [21]. In brief, the patient population had a mean±sd age of 48.5±13.3 years, 59% were female and the mean±sd EGPA duration was 5.5±4.6 years. Baseline characteristics were not notably different between mepolizumab and placebo groups (table 1).

**TABLE 1:** Demographics and baseline characteristics

	**Total**	**Mepolizumab 300 mg *s.c.***	**Placebo**
**Subjects, n**	136	68	68
**Age, years**	48.5±13.3	48.7±12.4	48.2±14.3
**Female**	80 (59)	42 (62)	38 (56)
**EGPA duration, years**			
Mean±sd	5.5 (4.6)	5.2 (4.4)	5.9 (4.9)
Median (range)	4.2 (0.5–25.9)	4.0 (0.7–25.9)	4.6 (0.5–21.2)
**History of refractory disease** **^#^**	74 (54)	34 (50)	40 (59)
**History of relapsing disease** **^#^**	100 (74)	51 (75)	49 (72)
**Number of relapses in the previous 2 years**			
0	5 (4)	2 (3)	3 (4)
1–2	73 (54)	42 (62)	31 (46)
≥3	57 (42)	24 (35)	33 (49)
**Baseline OCS dose, mg·day^−1^**	14.9±8.1	14.8±7.5	15.0±8.6
**Baseline immunosuppressant use**	72 (53)	41 (60)	31 (46)
Azathioprine	30 (22)	20 (29)	10 (15)
Methotrexate	24 (18)	13 (19)	11 (16)
Mycophenolic acid	12 (9)	6 (9)	6 (9)
Cyclosporine	3 (2)	0	3 (4)
Hydroxycarbamide	2 (1)	0	2 (3)
Leflunomide	2 (1)	1 (1)	1 (1)
Mycophenolate mofetil	1 (<1)	1 (1)	0

Data presented as n (%) or mean±sd, unless otherwise indicated. Due to data rounding, percentages may not add up to 100%. EGPA: eosinophilic granulomatosis with polyangiitis; OCS: oral corticosteroid; *s.c.*: subcutaneous. ^#^: patients could have either or both refractory/relapsing disease.

### Remission outcomes

Accrued duration of remission and the proportion of patients in remission at weeks 36 and 48 were greater with mepolizumab compared with placebo across the baseline clinical characteristics categories of refractory disease status, immunosuppressant use, EGPA duration and number of relapses, and in OCS dose baseline subgroups ≤20 mg·day^−1^ (table 2).

**TABLE 2:** Remission end-points by subgroup

**Subgroup**		**In remission at** **weeks 36 and 48**		**Accrued duration of remission** **^#^**
		**n (%)**	**OR (95 CI)** **^¶^** **(mepolizumab/placebo)**	**OR (95% CI)** **(mepolizumab/placebo)**
**Baseline refractory disease (within 6 months prior to screening)**				
With	Mepolizumab (n=34)	8 (24)	10.18 (1.15–90.15)	3.70 (1.29–10.65)
	Placebo (n=40)	1 (3)		
Without	Mepolizumab (n=34)	14 (41)	23.03 (2.51–211.47)	9.25 (2.44–35.08)
	Placebo (n=28)	1 (4)		
**Baseline immunosuppressant use**				
With	Mepolizumab (n=41)	13 (32)	7.12 (1.32–38.42)	3.39 (1.11–10.38)
	Placebo (n=31)	2 (6)		
Without	Mepolizumab (n=27)	9 (33)		11.85 (3.50–40.13)
	Placebo (n=37)	0		
**Baseline EGPA duration**				
≤4 years	Mepolizumab (n=34)	8 (24)		17.08 (3.41–85.54)
	Placebo (n=32)	0		
>4 years	Mepolizumab (n=34)	14 (41)	16.74 (3.05–91.96)	4.26 (1.53–11.91)
	Placebo (n=36)	2 (6)		
**Number of relapses in the previous 2 years**				
0	Mepolizumab (n=2)	0		
	Placebo (n=3)	0		
1–2	Mepolizumab (n=42)	13 (31)	6.91 (1.25–38.35)	2.60 (0.86–7.85)
	Placebo (n=31)	2 (6)		
≥3	Mepolizumab (n=24)	9 (38)		15.98 (4.10–62.25)
	Placebo (n=33)	0		
**Baseline OCS dose, mg·day^−1^**				
≤7.5	Mepolizumab (n=6)	5		34.16 (1.95–599.71)
	Placebo (n=12)	0		
>7.5 –≤12	Mepolizumab (n=31)	10	13.35 (1.34–133.28)	6.66 (1.91–23.24)
	Placebo (n=2)	1		
>12–≤20	Mepolizumab (n=20)	7		17.91 (2.88–111.45)
	Placebo (n=22)	0		
>20	Mepolizumab (n=11)	0		0.72 (0.07–7.40)
	Placebo (n=10)	1		

EGPA: eosinophilic granulomatosis with polyangiitis; OCS: oral corticosteroid. ^#^: categories are 0 weeks, >0–11 weeks, 12–23 weeks; 24–35 weeks, ≥36 weeks; ^¶^: OR for remission at weeks 36 and 48 could not be calculated when there were no patients in the placebo arm in remission.

Specifically, patients with and without refractory disease within 6 months of baseline spent more weeks in remission across the duration of the study with mepolizumab compared with placebo (with refractory disease: OR 3.70, 95% CI 1.29–10.65; without refractory disease: OR 9.25, 95% CI 2.44–35.08), and were more likely to be in remission at weeks 36 and 48 of the trial with mepolizumab compared with placebo (mepolizumab: OR 10.18, 95% CI 1.15–90.15; placebo: OR 23.03, 95% CI 2.51–211.47). Similarly, patients with and without baseline immunosuppressant use accumulated more weeks in remission with mepolizumab compared with placebo (immunosuppressant use: OR 3.39, 95% CI 1.11–10.38; no immunosuppressant use: OR 11.85, 95% CI 3.50–40.13). Patients also accumulated more weeks in remission with mepolizumab compared with placebo regardless of EGPA duration (EGPA duration ≤4 years: OR 17.08, 95% CI 3.41–85.54; EGPA duration >4 years: OR 4.26, 95% CI 1.53–11.91).

More patients treated with mepolizumab compared with placebo were in remission at weeks 36 and 48 across baseline immunosuppressant use and EGPA duration subgroups (table 2). Across subgroups of patients with a history of 1–2 or ≥3 relapses in the 2 years before enrolment, remission end-points favoured mepolizumab compared with placebo. Patients treated with mepolizumab spent more time in remission compared with patients treated with placebo in both the 1–2 relapse and ≥3 relapse subgroups (1–2 relapse: OR 2.60, 95% CI 0.86–7.85; ≥3 relapse: OR 15.98, 95% CI 4.10–62.25), and were more likely to be in remission at weeks 36 and 48 (table 2). There were insufficient data to analyse remission outcomes in patients with no relapses in the previous 2 years owing to small patient numbers in the mepolizumab (n=2) and placebo (n=3) subgroups, none of whom achieved remission by weeks 36 and 48 (table 2). Analysis of duration of remission and the number of patients in remission at weeks 36 and 48 by baseline OCS dose favoured mepolizumab treatment for all subgroups of OCS doses ≤20 mg·day^−1^, but not for the OCS dose >20 mg·day^−1^ subgroup, for which the overall number of patients achieving any remission during the treatment period or remission at both weeks 36 and 48 was small (n=5 and n=1, respectively; table 2).

### Clinical benefit

**FIGURE 1:**
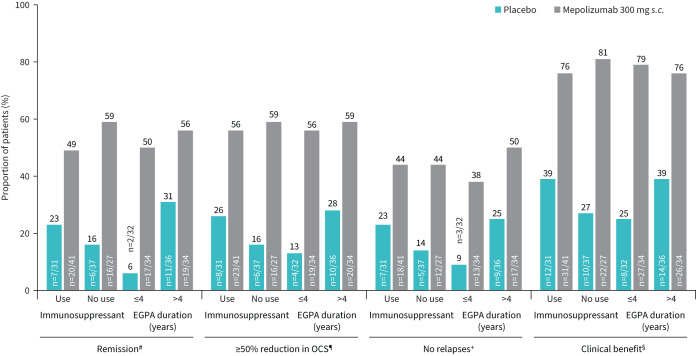
Proportion of patients with clinical benefit by immunosuppressive therapy and eosinophilic granulomatosis with polyangiitis (EGPA) duration subgroups. BVAS: Birmingham Vasculitis Activity Score; OCS: oral corticosteroid; *s.c.*: subcutaneous. ^#^: BVAS=0 and prednisolone/prednisone dose ≤4 mg·day^−1^ at any time; ^¶^: prednisolone/prednisone dose during weeks 48–52; ^+^: no EGPA relapses during the treatment period; ^§^: remission and/or ≥50% reduction in OCS and/or no relapses.

Numerically, more patients achieved remission, achieved ≥50% reduction in OCS dose during weeks 48–52 and experienced no relapses during the study (each considered a clinical benefit) with mepolizumab than with placebo regardless of baseline immunosuppressant use or EGPA duration (figure 1). Additionally, more patients experienced one or more of these three clinical benefits with mepolizumab compared with placebo (immunosuppressant use: 76% *versus* 39%; no immunosuppressant use: 81% *versus* 27%; EGPA duration ≤4 years: 79% *versus* 25%; EGPA duration >4 years: 76% *versus* 39%; figure 1).

### OCS-sparing effects

**FIGURE 2:**
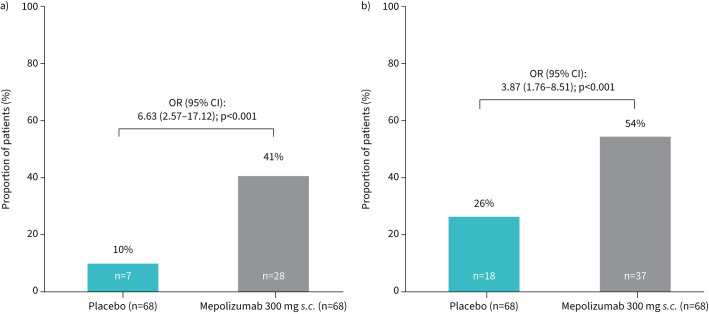
Proportion of patients with oral corticosteroid dose a) ≤4 mg·day^−1^ and b) ≤7.5 mg·day^−1^ at weeks 36 and 48. *s.c.*: subcutaneous.

The proportion of patients achieving ≥50% reduction in OCS dose during weeks 48–52 was numerically greater with mepolizumab compared with placebo across baseline immunosuppressant use (immunosuppressant use: 56% *versus* 26%; no immunosuppressant use: 59% *versus* 16%) and disease duration subgroups (EGPA duration ≤4 years: 56% *versus* 13%; EGPA duration >4 years: 59% *versus* 28%). In the overall study population, the proportion of patients receiving an OCS dose ≤4 mg·day^−1^ at week 36 and 48 was significantly greater with mepolizumab (41%, n=28 out of 68) compared with placebo (10%, n=7 out of 68; OR 6.63, 95% CI 2.57–17.12, p<0.001; figure 2a). Similarly, use of ≤7.5 mg·day^−1^ OCS was significantly greater with mepolizumab (54%, n=37 out of 68) compared with placebo (26%, n=18 out of 68; OR 3.87, 95% CI 1.76–8.51, p<0.001; figure 2b).

**FIGURE 3:**
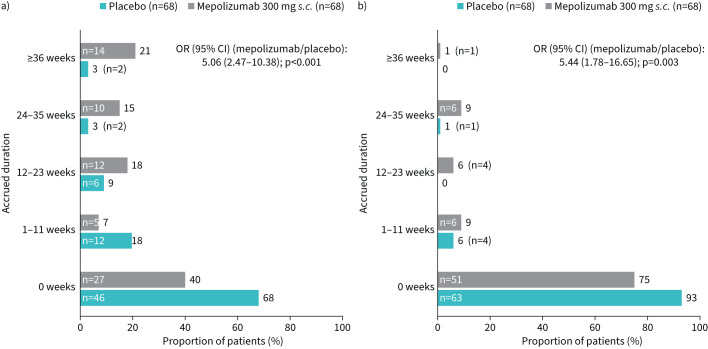
Accrued duration of oral corticosteroid dose a) ≤4 mg·day^−1^ and b) =0 mg·day^−1^ over the 52-week treatment period. *s.c.*: subcutaneous.

Patients treated with mepolizumab compared with placebo accrued more weeks on ≤4 mg·day^−1^ over the 52-week study period (OR 5.06, 95% CI 2.47–10.38, p<0.001; figure 3a). For instance, 21% of patients treated with mepolizumab accrued ≥36 weeks of OCS ≤4 mg·day^−1^ compared with 3% on placebo. More patients (25%, n=17) achieved OCS-free weeks when treated with mepolizumab compared with placebo (7%, n=5; figure 3b).

**FIGURE 4:**
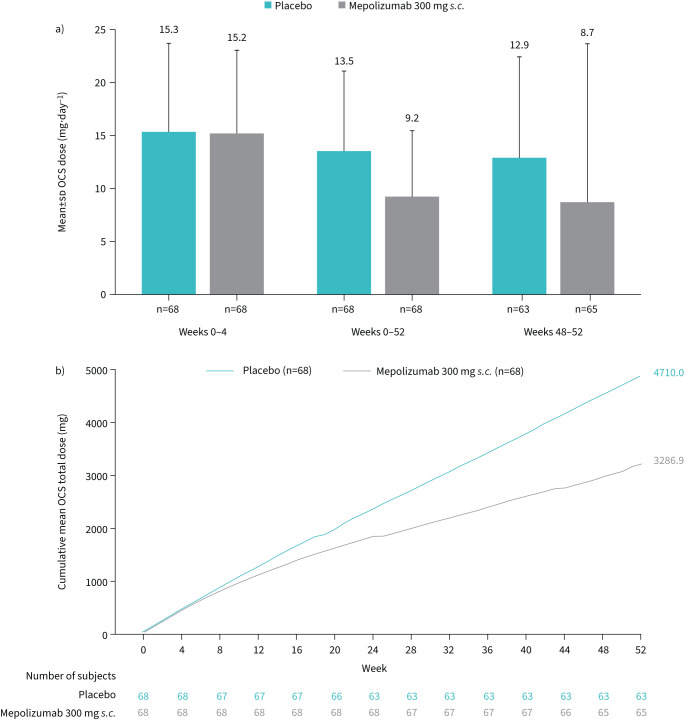
a) Mean daily oral corticosteroid (OCS) dose during weeks 0–4, weeks 0–52 and weeks 48–52. b) Mean cumulative OCS exposure over the 52-week study period. In assessing cumulative dose, observed data following treatment discontinuation were used where available for participants who withdrew from treatment. *s.c.*: subcutaneous.

By the final 4 weeks of the study period, both the mean and median daily OCS dose were lower with mepolizumab compared with placebo (mean±sd 8.7±14.9 mg·day^−1^ *versus* 12.9±9.5 mg·day^−1^; median (minimum–maximum): 5.0 mg·day^−1^ (0–113.4 mg·day^−1^) *versus* 10.0 mg·day^−1^ (0–46.3 mg·day^−1^), respectively; figure 4a). There was also a substantial difference in the mean cumulative total OCS received over the study period, with patients treated with mepolizumab (3286.9 mg) receiving on average 1423.1 mg less than patients treated with placebo (4710.0 mg) over the 52-week study period, which is equivalent to 4.3 mg less per day (figure 4b, c).

## Discussion

These *post hoc* analyses aimed to further characterise whether baseline variables predict response to mepolizumab and to provide new details on the corticosteroid-sparing effects of mepolizumab in patients with EGPA. Patients treated with mepolizumab had a longer accrued duration of remission and more patients were in remission at weeks 36 and 48 compared with placebo across subgroups of baseline refractory disease status, immunosuppressant use, EGPA duration, number of relapses and OCS use ≤20 mg·day^−1^. In addition, regardless of immunosuppressant use and disease duration, patients treated with mepolizumab were more likely to receive clinical benefits in remission, reduced OCS exposure and reduced rates of relapse. Furthermore, patients treated with mepolizumab compared with placebo experienced a clinically meaningful lower cumulative OCS exposure across the treatment period and accumulated more OCS-free weeks. Together, these results indicate that patients with relapsing or refractory EGPA are likely to receive clinical benefit from mepolizumab treatment regardless of disease and treatment history, and that mepolizumab should be considered as an OCS-sparing treatment.

The present analyses further investigated the pattern of OCS-sparing outcomes in the total study population, and added new details to the previously reported outcome of OCS reductions in the primary clinical trial results [21]. The importance of minimising OCS exposure in patients with EGPA is demonstrated by the burden of OCS-related adverse effects for patients with other chronic diseases. OCS use in patients with asthma and patients with giant cell arteritis leads to an increased risk of both acute and chronic complications, which increases with OCS dose [12, 14, 29–31]. For example, for patients with giant cell arteritis, every 1000 mg of total OCS exposure increases the risk of a first adverse event by 3% [31]. The >1400 mg reduction in OCS exposure with mepolizumab compared with placebo over the 52-week MIRRA study period is therefore likely to be of clinical significance.

Similarly, a European Alliance of Associations for Rheumatology task force found that the risks of long-term OCS exposure were more pronounced at doses >5 mg·day^−1^; risks increased further at >10 mg·day^−1^ and may outweigh the benefits for many patients [15]. The present analysis revealed that patients treated with mepolizumab compared with placebo were more likely to have their OCS dose reduced to ≤4 or ≤7.5 mg·day^−1^ at weeks 36 and 48 and accrued more weeks of OCS ≤4 mg·day^−1^ throughout the treatment period. Furthermore, 25% of patients receiving mepolizumab accumulated OCS-free weeks. The OCS-sparing effects demonstrated in the MIRRA study are therefore likely to have a considerable impact on the OCS-related burden experienced by patients [14, 16].

The rationale for investigating the potential for clinical characteristics to influence response to mepolizumab arose from the variability in treatment response to mepolizumab seen in MIRRA, with 47% of patients treated with mepolizumab not achieving protocol-defined remission (compared with 81% of the placebo group). Many prior studies of mepolizumab and other anti-IL-5 therapeutics for severe eosinophilic asthma have demonstrated that higher eosinophil counts (≥150 cells·µL^−1^) predict greater treatment response [17]. This trend was also seen for patients with EGPA during MIRRA; patients treated with mepolizumab with blood eosinophil counts ≥150 cells·µL^−1^ had an increased accrued duration of remission, whereas this benefit, while still positive, was less robust in patients with blood eosinophil count <150 cells·µL^−1^ [21].

Identifying the characteristics of those patients with EGPA who may be more likely to respond to mepolizumab is important to optimise treatment. Five clinical characteristics with the potential to influence treatment outcomes for the patient were investigated in our *post hoc* re-analysis of MIRRA co-primary outcomes: baseline refractory disease status, immunosuppressant use, OCS dose, EGPA duration and number of previous relapses. Refractory disease, immunosuppressant use and high OCS use all suggest difficult-to-control disease, while longer disease duration and more previous relapses could indicate greater cumulative organ or tissue damage. Of these characteristics, only patients receiving the highest baseline OCS dose (>20 mg·day^−1^) had suboptimal remission as compared with all other subgroups. The necessity for gradual tapering of OCS made it more challenging for patients starting on the highest OCS doses to achieve protocol-defined remission outcomes within the study timeframe. Both accrued time in remission and the proportion of patients in remission at weeks 36 and 48 favoured mepolizumab compared with placebo in all other subgroups. The benefits of mepolizumab in broader populations of patients with EGPA have also been demonstrated in real-world studies [26–28, 32]. For example, an ongoing mepolizumab post-marketing surveillance study in Japan reported improved clinical symptoms, blood eosinophil count and OCS use by week 48 compared with baseline levels [33]. Despite recent advances in treatment options for patient with EGPA, unmet needs remain and future research should include a focus on identifying predictors of response to mepolizumab, delineating the optimal combinations of therapies to improve outcomes, and quantifying the effects of mepolizumab treatment on health-related quality of life [34–36].

There are several limitations which need to be considered when interpreting these results. Results presented here are *post hoc* analyses of phase 3 clinical trial data in a rare disease. As such, the number of patients in some subgroups was small and the subgroup analyses were not powered for statistical testing. Additionally, the population of patients with EGPA included in this trial was limited by the requirement for each patient to be taking a stable dose OCS ≥7.5–≤50 mg·day^−1^ at baseline. Therefore, benefits in a broader population including patients on unstable, low or very high OCS doses were not investigated. Additionally, EGPA is a rare and heterogenous disease with no consensus diagnostic criteria and limited tools available to capture EGPA disease activity. The definitions of relapse and remission used in the MIRRA study and the present analysis are dependent on the BVAS, a tool developed to characterise vasculitis, which is not specific to EGPA.

### Conclusion

Remission-related outcomes favoured mepolizumab over placebo across baseline subgroups of refractory disease status, immunosuppressant use, EGPA duration, number of previous relapses and OCS dose ≤20 mg·day^−1^. Mepolizumab also provides clinical benefits, including reduced OCS exposure, regardless of baseline immunosuppressant use or EGPA duration. In the overall population, greater reductions in OCS use were observed with mepolizumab compared with placebo at multiple timepoints and cumulatively. These results build on previous evidence of the benefits of mepolizumab in patients with EGPA and indicate that patients with EGPA with varying disease and treatment history are likely to have clinical benefits from mepolizumab treatment.

## Supplementary material

10.1183/23120541.00509-2023.Supp1 **Please note:** supplementary material is not edited by the Editorial Office, and is uploaded as it has been supplied by the author.
Supplementary material 00509-2023.SUPPLEMENT
