# Supplementary material for: Mepolizumab has clinical benefits including oral corticosteroid sparing irrespective of baseline EGPA characteristics
Source: ERJ Open Res. 2024 Jan 8;10(1):00509-2023. doi: 10.1183/23120541.00509-2023 (PMC10772899; doi:10.1183/23120541.00509-2023)
Supplement: Supplementary file 1 [file 00509-2023.SUPPLEMENT.pdf]

Online Supplement

Mepolizumab Has Clinical Benefits Including OCS Sparing Irrespective of Baseline EGPA Characteristics

David RW Jayne FMedSci, Benjamin Terrier MD PhD, Bernhard Hellmich MD, Paneez Khoury MD MHS, Lee Baylis MD, Jane H Bentley MSc, Jonathan Steinfeld MD, Steven W Yancey MSc, Namhee Kwon MD, Michael E Wechsler MD, Praveen Akuthota MD, on behalf of the EGPA mepolizumab study team

EGPA mepolizumab study team:

- Chiara Baldini, Centro Farmacologia Clinica AOUP, Pisa, Italy; Douglas M Beach, Beth Israel Deaconess Medical Center, Boston, MA, USA;
- Bruce Bochner, Northwestern University; Arnaud Bourdin, Hôpital Arnaud de Villeneuve, Montpellier, France;
- Pascal Chanez, Hôpital Nord, Marseille, France; Anoop Chauhan, Queen Alexandra Hospital, Portsmouth, UK; Vincent Cottin, Hôpital Louis Pradel, Bron, France;
- Maria C Cid, the Vasculitis Research Unit, Department of Autoimmune Diseases, Hospital Clinic University of Barcelona, Institut d'Investigacions Biomèdiques August Pi i Sunyer (IDIBAPS), Barcelona, Spain;
- Bertrand Dunogue, Hôpital Cochin, Paris, France;
- Georgina Espígol-Frigolé, Department of Autoimmune Diseases, Hospital Clínic University of Barcelona, IDIBAPS, Barcelona, Spain;
- Aryeh Fischer, National Jewish Health, Denver, CO, USA;
- Gerald J Gleich, the Departments of Dermatology and Medicine, University of Utah School of Medicine, Salt Lake City, UT, USA;
- Rula Hajj-Ali, Cleveland Clinic, Cleveland, OH, USA;
- Berhnard Hellmich, Kreiskliniken Esslingen Klinik Kirchheim, Kirchheim unter Teck, Germany;
- José Hernández Rodríguez, Department of Autoimmune Diseases, Hospital Clínic University of Barcelona, Institut d'Investigacions Biomèdiques August Pi I Sunyer (IDIBAPS), Barcelona, Spain;
- Julia Holle, Rheumazentrum Schleswig-Holstein Mitte, Neumünster Germany;
- Christof Iking-Konert, Rheumaklinik Bad Bramstedt & Universitaetsklinikum Schleswig-Holstein, Bad Bramstedt, Germany;
- Tomonori Ishii, Tohoku University Hospital, Miyagi, Japan;
- Jean-Emmanuel Kahn, Hôpital Foch, Suresnes, France; Peter Kern, Klinikum Fulda-MVZ Osthessen, Hessen, Germany;
- Nader Khalidi, St. Joseph's Healthcare Hamilton, Hamilton, ON, Canada;
- Amy Klion, the Human Eosinophil Section, Laboratory of Parasitic Diseases, National Institute of Allergy and Infectious Diseases, National Institutes of Health, Bethesda, MD;
- Curry L Koening, Department of Medicine, University of Utah, School of Medicine, Salt Lake City, UT, USA;
- Carol A Langford, the Center for Vasculitis Care and Research, Cleveland Clinic, Cleveland, USA;

- Andrea Matucci, Azienda Ospedaliero-Universitaria di Careggi, Firenze, Italy;
- Peter A. Merkel, the Division of Rheumatology and the Department of Biostatistics and Clinical Epidemiology, University of Pennsylvania; USA;
- Frank Moosig; Rheumazentrum, Schleswig–Holstein Mitte, Neumünster, Germany;
- Paul Monach, Boston University School of Medicine, Boston, MA, USA;
- Thomas Neumann, Klinikum der Friedrich Schiller Universitaet Jena, Thueringen, Germany;
- Christian Pagnoux, Mount Sinai Hospital, Toronto, ON, Canada;
- Elide Pastorello, Azienda Ospedaliera Ospedale Niguarda Cà Granda, Milano, Italy;
- Sergio Prieto-González, Department of Autoimmune Diseases, Hospital Clínic University of Barcelona, Institut d'Investigacions Biomèdiques August Pi I Sunyer (IDIBAPS), Barcelona, Spain;
- Xavier Puechal, Hôpital Cochin, Paris, France;
- Benjamin A. Raby, Brigham & Women's Hospital, Boston, MA, USA;
- Florence Roufosse, Hôpital Erasme, Bruxelles, Belgium; Maria Grazia Sabbadini, Ospedale San Raffaele IRCCS, Milano, Italy;
- Jan Schirmer, University Hospital of Kiel, Kiel, Germany;
- Ulrich Specks; the Division of Pulmonary and Critical Care Medicine, Mayo Clinic, Rochester, MN, USA;
- Antoine G Sreih, University of Pennsylvania, Philadelphia, PA, USA;
- Masami Taniguchi, Sagamihara National Hospital, Kanagawa, Japan; Reinhard Voll, Universitaetsklinikum Freiburg, Freiburg, Germany;
- Andrew Wardlaw, Respiratory Biomedical Research Unit, Glenfield Hospital, Leicester, UK;
- Peter F Weller, Beth Israel Deaconess Medical Center, Boston, MA, USA.
